# A Successful Collagenase and Hyaluronic Acid Topical Use Combined with Antibiotic Therapy in the Treatment of Ulcerative Lesions Arising on Tattoo

**DOI:** 10.1155/2012/253492

**Published:** 2012-11-11

**Authors:** Paolo Fioramonti, Pasquale Fino, Martina Ruggieri, Nicolò Scuderi, Maria Giuseppina Onesti

**Affiliations:** Department of Plastic, Reconstructive and Aesthetic Surgery, Sapienza University of Rome, Policlinico Umberto I, Viale del Policlinico, 155, 00161 Rome, Italy

## Abstract

Tattooing is an ancient art widely practiced allover the world. The procedure used in making a tattoo consists of puncturing the dermis and depositing an amount of ink, but this decorative technique is not devoid of potential adverse effects, such as infectious complications, hypersensitivity reactions to pigments (lichenoid and granulomatous reactions), the development of benign or malignant tumors, and chronic dermatoses on site of the tattoo. We report an unusual complication which arose on a colorful tattoo and the innovative use of collagenase (Bionect Start) to treat it. With conservative treatment and the use of Bionect Start, we obtained complete resolution and reepithelialization of the lesions, in addition to perfect preservation of the colors of the tattoo.

We present an unusual complication arose on a tattoo and the innovative use of collagenase to treat it. Tattooing is an ancient art and is still widely practiced all over the world. After the procedure, a wound healing process starts with an instantaneously acute inflammatory phase, followed by a proliferative and maturation phase to restore tissue integrity [1]. Skin complications include infections, hypersensitivity reactions to pigments, chronic dermatoses, and the development of benign or malignant tumors on site of tattoo [2].

A 25-year-old man, two weeks after making a colorful tattoo on his right arm, showed the development of four ulcerative lesions on it. They were about 0.5–1.0 cm in diameter, oval in shape, with regular margins and net limits; there was an aura surrounding erythematosus; the bottom of the lesion was covered by purulent discharge and abundant fibrin (Figure 1). Furthermore, the patient referred locoregional pain, itching, and heat. A swab of wound secretions was performed and it resulted positive for *Staphylococcus aureus* and *Streptococcus pyogenes*. So we have given the patient oral antibiotic therapy based on clavulanic acid 875 mg and amoxicillin 125 mg (2 tablets/day for 6 days). We also performed daily medication of ulcerative lesions disinfecting them with sodium hypochlonte 0.05% cutaneous solution (Amukine Med 0.05%, Amuchina S.P.A genova-Italy), cutaneous solution based on 10% povidone iodine (Betadine 10%, Meda Pharma S.P.A Milano-Italy), and applying a film of about 2 mL of Bionect Start (Fida Pharmaceutical, Abano Treme-Italy) ointment on the lesions and zinc oxide paste onto the surrounding skin. Bionect Start is a topical cream containing hyaluronate acid, bacterial fermented sodium hyalunorate (0.2% w/w) salt, and bacterial collagenase obtained from nonpathogenic Vibrio alginolyticus (>2.0 nkat1/g) [3]. The use of collagenase is based on performing lysis of fibrin and necrotic tissue. The topical administration of collagenase increases the effect of macrophage collagenase, which is responsible for wound debridement by splitting and breaking down proteins that hold the eschar (dead and devitalised material) over the wound [1, 3]. Bionect Start is shown to provide an optimal moist environment and a wound preparation to facilitate the natural healing process [4]. This drug also contains hyaluronic acid (HA) which above all generates a microenvironment stimulating the secretion of growth factors, proliferation and migration of fibroblasts, endothelial cells, keratinocytes, and angiogenesis and it has a positive effect on inflammatory response [1, 4, 5]. Moreover, HA is also capable of regulating the water balance acting on osmotic pressure and flow resistance and selectively sieving the diffusion of plasma and matrix proteins [5]. So, after 7 days of conservative treatment with the use of Bionect Start, we obtained resolution of symptoms and completed reepithelialization of the lesions (Figure 2).

The microbiological test result allowed us to give the patient a specific antibiotic therapy to treat the infection on the tattoo. Probably the wound healing was also possible with the simple cleansing, disinfection, and antibiotic therapy without the use of collagenase, but in that case the scar would be disfiguring with unsatisfactory aesthetic results. So, the use of this collagenase has allowed us to achieve good scars, and it has mainly allowed to keep the colors of the tattoo, requiring no editing after healing (Figure 2).

## Figures and Tables

**Figure 1:**
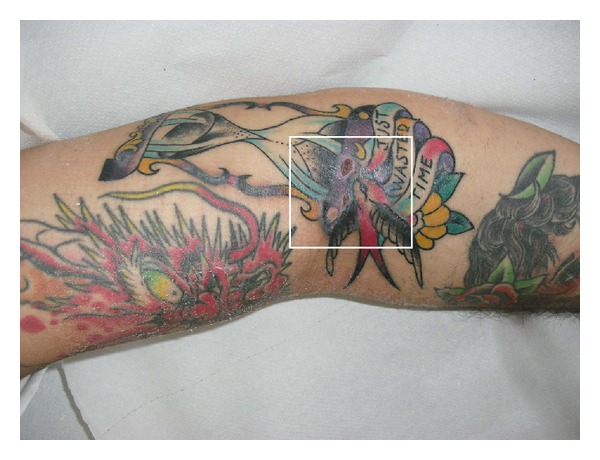
Ulcerative lesions of the right arm (shown in the square).

**Figure 2:**
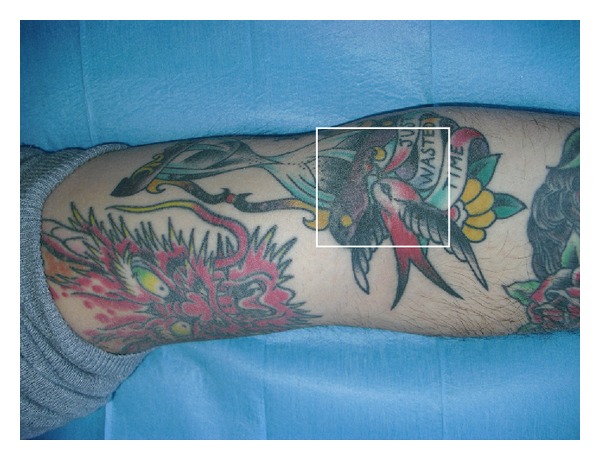
Complete reepithelialization of the lesions of the right arm after 7 days (shown in the square).
